# Developing a bioethics curriculum for medical students from divergent geo-political regions

**DOI:** 10.1186/s12909-016-0711-4

**Published:** 2016-07-27

**Authors:** Rebecca A. Greenberg, Celine Kim, Helen Stolte, Jonathan Hellmann, Randi Zlotnik Shaul, Rahim Valani, Dennis Scolnik

**Affiliations:** 1 Department of Bioethics, The Hospital for Sick Children, Toronto, ON Canada; 2 Division of Emergency Medicine, Health Sciences Centre, McMaster University, Hamilton, ON Canada; 3 Osgoode Hall Law School, University of Toronto, Toronto, ON Canada; 4 Department of Bioethics, The Hospital for Sick Children, University of Toronto, Toronto, ON Canada; 5 Divisions of Paediatric Emergency Medicine and Clinical Pharmacology & Toxicology, The Hospital for Sick Children, University of Toronto, Toronto, ON Canada

## Abstract

**Background:**

The World Health Organization calls for stronger cross-cultural emphasis in medical training. Bioethics education can build such competencies as it involves the conscious exploration and application of values and principles. The International Pediatric Emergency Medicine Elective (IPEME), a novel global health elective, brings together 12 medical students from Canada and the Middle East for a 4-week, living and studying experience. It is based at a Canadian children’s hospital and, since its creation in 2004, ethics has informally been part of its curriculum. Our study sought to determine the content and format of an ideal bioethics curriculum for a culturally diverse group of medical students.

**Methods:**

We conducted semi-structured interviews with students and focus groups with faculty to examine the cultural context and ethical issues of the elective. Three areas were explored: 1) Needs Analysis - students' current understanding of bioethics, prior bioethics education and desire for a formal ethics curriculum, 2) Teaching formats - students’ and faculty’s preferred teaching formats, and 3) Curriculum Content - students’ and faculty’s preferred subjects for a curriculum.

**Results:**

While only some students had received formal ethics training prior to this program, all understood that it was a necessary and desirable subject for formal training. Interactive teaching formats were the most preferred and truth-telling was considered the most important subject.

**Conclusions:**

This study helps inform good practices for ethics education. Although undertaken with a specific cohort of students engaging in a health-for-peace elective, it may be applicable to many medical education settings since diversity of student bodies is increasing world-wide.

**Electronic supplementary material:**

The online version of this article (doi:10.1186/s12909-016-0711-4) contains supplementary material, which is available to authorized users.

## Background

Historically, medical graduates have tended to practice close to their place of training, focusing on local needs [1]. Recently however, globalization and immigration have dictated a more ‘global’ appreciation of differences in the practice of medicine across borders and internationally. This is reflected in medical school classes where students from different cultural, religious, and geopolitical backgrounds are now training side by side [1]. In addition, medical trainees are increasingly choosing to participate in international medical electives, often in developing countries, [1] for example, in 2010, 30 % of United States medical graduates participated in international health experiences compared to 6 % in 1984 [2, 3]. Individuals from different cultures may have disparate approaches in the clinical application of medical bioethics, despite subscribing to similar core values. During global health electives these differences can pose ethical problems if not addressed [4]. Since many core bioethical principles have roots in cultural, rather than universal norms, clinical ethical practice is not uniform, and teaching bioethics to a diverse group poses unique challenges [5]. Today ‘diversity is the norm and not the exception’ [6] and teaching in bioethics remains unduly influenced by the misconception of homogeneity in medical student body composition [7]. This can lead ethics educators to neglect important differences in the moral understanding of different religious and ethnic groups [7] and suboptimal healthcare outcomes that may involve matters of life and death [8]. Unresolved ethics issues can also have a detrimental impact on both the quality of patient care and the culture of healthcare organizations [9]. Bioethics education must be mindful, from both content and methodological perspectives, of the presumed values it presents and imposes upon the learner. It should recognize and understand cultural context to determine the methods most conducive to achieving desired educational outcomes. To optimize learning outcomes and address challenges in diverse group-learning environments it is crucial that educators appreciate issues relating to group composition and dynamics [10]. Medical educators have called for greater curricular allocation to ‘the wide range of cultural, environmental and ethical issues that will increasingly impinge on the problems of health’ [11]. Training in bioethics should enhance physicians’ ability to navigate culturally sensitive ethical issues and improve quality of patient care; however, there is currently a paucity of information about how bioethics should be taught across cultural boundaries [12].

The International Pediatric Emergency Medicine Elective (IPEME) is an innovative ‘peace-through-health’ initiative at the University of Toronto and McMaster University in Canada. Introduced in 2004, its underlying goal is to increase cross-border cooperation among medical students from different geopolitical backgrounds. During this four week elective, pediatric emergency medicine constitutes the common vehicle that drives other components of the program such as leadership, conflict resolution, global health, clinical competence and research. Students also spend time on a research project, working in groups, with one student from each region in each group. Catering to such a culturally diverse group of medical students IPEME provides a unique opportunity to gain a broader understanding of pediatric bioethics in the context of trainees from different backgrounds. During the early years of the program, no formal bioethics teaching was incorporated in the curriculum. Given the importance of bioethics education, and the lack of a specific curriculum that reaches across geopolitical divides, a project was designed to develop such a curriculum. We set about trying to answer the following questions: How could we develop a curriculum that explored influences on bioethics that have roots in cultural rather than ethical norms as opposed to those that are more universal. What teaching content and methods best facilitate this and why is this so? We hoped that our findings could be generalized to the mixed cultural settings that are becoming the norm rather than the exception in many health care settings.

## Methods

This was a qualitative study approved by the Research Ethics Board, The Hospital for Sick Children, University of Toronto, Ontario, Canada.

### Population

The 2013 IPEME student cohort was approached and asked to participate in an individual, semi-structured interview exploring their experience with bioethics teaching. To help account for this being a study based on a single cohort of medical students, faculty views were sought to reflect general historic views on the elective. A focus group was also held with four IPEME faculty to identify and examine any longstanding ethical issues encountered during the elective over previous years. Finally, to explore cultural context and ethical issues, individual interviews were held with four members of the University of Toronto Joint Centre for Bioethics (Additional file 3: Appendix 3 and see Endnotes) engaged in educating culturally diverse groups.

### Setting

A total of 30–40 applications is received annually, from any of the medical schools in the different regions. Middle Eastern students are in their final two years of a six-year medical education and have all had a substantial amount of patient contact. Canadian medical students are in the first two years of a four-year medical education and most have had only limited direct patient contact. The application process requires the candidate to write about their involvement with cross-border issues, leadership and global health. Applications are reviewed and scored by a program co-director and an alumnus (a). The final cohort consists of three Canadian, three Israeli, three Jordanian and three Palestinian medical students. The setting in Toronto provides a safe space to facilitate interactions between participants. Potential economic barriers to attendance are obviated by all travel, accommodation, food and visa costs being fully subsidized. Participants are provided a room in a common university residence and eat and spend each work day, and much of their casual time, with one another, to potentiate communication and sharing of experiences.

For each of their three daily learning sessions, students are accompanied by a senior pediatric trainee with a special interest in promulgating the aims of the program, facilitating and modelling dialogue and identifying key issues requiring the intercession of senior faculty. IPEME co-directors are a readily available resource and meet with students many times a week.

### Data collection

For students, an individual 30-min, semi-structured interview was conducted by a research assistant not known to the students, to increase comfort levels of participants and limit bias or leading. Six open-ended questions were asked (Additional file 1: Appendix I). Three specific areas were addressed: 1) Needs analysis – an exploration of students’ understanding of bioethics, prior bioethics education and the desire for a formal ethics curriculum, 2) Teaching formats – an exploration of students’ and faculty’s preferred teaching format, and 3) Curriculum content - an exploration of students’ and faculty’s preferred subjects to comprise a curriculum. Interviews and focus groups were audio-recorded and transcribed. The content of the semi-structured interview was informed by ethics encounters with, and between, students from previous IPEME cohorts.

Questions guiding focus group discussion with IPEME faculty are shown in Additional file 2: Appendix II and those used for individual interviews with University of Toronto Joint Centre for Bioethics faculty in Additional file 3: Appendix III. The latter examined the relevance of a bioethics curriculum for IPEME and similar programs, common ethical issues of relevance to the program, and best teaching formats for delivery of such a curriculum.

### Data analysis

Interviews and focus groups were transcribed and analyzed in three phases: open coding, axial coding, and evaluation. In open coding the full transcript was read and fractured into sections by identifying key concepts or codes e.g. sexual health. In axial coding transcripts were then examined together and their concepts were compared and organized into broader themes e.g. gender issues. In the evaluation phase the data set was reviewed multiple times to allow comparison within and between interviews and focus groups. Points of agreement between student and faculty were noted as especially relevant areas for curriculum development. Key themes were used to help identify both the content of the ethics curriculum e.g. gender issues, allocation of resources etc. and the appropriate delivery format e.g. role play, lectures etc. To ensure consistency and accuracy, two researchers separately coded the data and then discussed their findings to establish areas of convergence or divergence. Where disagreement arose, further discussion and referencing of the literature were used to find agreement.

## Results

Of the 12 students in the cohort 10 consented to be interviewed.

### Needs analysis

#### Understanding of Bioethics

Most students showed a general appreciation of bioethics, understanding it to be a discipline employing a broad set of principles to help inform decision-making. Some students believed that there was a universal standard or consensus for ethical decision making, citing ethics to be:

“An instrument that can help you choose between right and wrong.”

“A universal standard or code that helps to inform difficult decisions.”

#### Prior ethics training

Most students reported little or no formal ethics teaching. Ethics was commonly taught informally through modeling, by mentors. Some students did have a structured bioethics curriculum as part of their training and one participant mentioned having access to a bioethicist to consult regarding ethical dilemmas.

Students used religion as a framework to guide ethical decision-making, a sentiment echoed by faculty as a tool used by previous cohorts:

“[I look to] what the religion says about dilemmas.”

#### Need for curriculum

Both students and faculty were resoundingly in favor of a more formal curriculum, and increased ethics teaching around case-based scenarios. Students wanted more time to explore ethical topics in depth during IPEME. Faculty felt that a formal curriculum could offer several benefits, including the ability to teach certain topics in a more explicit manner:

“[I want to know] assumptions behind decisions … [and] awareness of what informs our decisions.”

### Teaching format

Students identified four teaching formats for optimal curriculum delivery: case-based format, role-play opportunities, video examples, and small group discussion.

#### Case-based

Many suggested that case studies were the most effective learning tool as they provided a more active means of thinking through a real situation, and constituted a more concrete learning tool compared with abstract theory or principles. Students wanted guidance regarding possible answers to bioethical scenarios presented.

#### Role-play

Many students suggested role-play as being a good way to demonstrate concepts learned in the course, or to re-enact encountered clinical scenarios.

#### Video

Several students mentioned the use of videos of patient encounters (real or simulated) as being a potentially valuable tool to demonstrate how to handle bioethical dilemmas.

#### Small group discussion

Students liked the idea of small group breakout sessions to discuss particular concepts or cases.

When asked what formats were least ideal, participants unanimously cited lecture-based slide presentations, however they still wanted foundational information and they recognized that slides might be the modality for this. A few participants stated that, although they valued wide-ranging discussion, it was important that discussions not to be too open-ended.

Faculty agreed that the students seemed engaged in case-based and role-play teaching formats, and perhaps less so in more structured lecture settings, but views diverged as to whether or not lectures were a valuable tool:

“I think most students and most faculty probably don’t like the classic talking head lecture … “

“Well, you need a foundation, you need the language.”

Faculty underscored the importance of selecting issues and topics that students prioritized and ensuring that a holistic perspective was maintained in delivering the curriculum:

“I find that there has been in the past a sense that we’re trying to kind of push our Westernized ethical views and perspectives onto students who don’t feel that it applies to them in their setting.”

### Curriculum content

Students and faculty identified five key topics for the curriculum.

#### Truth-telling

Truth-telling was, by far, the most important topic raised by students. They articulated that it was difficult, or sometimes inappropriate, to tell the truth. Within the concept of truth-telling, of particular concern was determining when it would be appropriate to disclose information to a child whose parents wanted to withhold information:

“It’s not in a bad way that the parents don’t tell the children what they have… They want to protect him, even from the bad information, they don’t want his psychology affected so it’s just their way of protecting that child.”

Additionally, exploring the ethics of disclosure of diagnosis to the elderly was a topic of interest:

“We have many cases [of cancer and] the patient they don’t know that they have cancer.”

#### Gender issues

Both students and faculty raised ethical issues related to gender:

“It gets really interesting, people get really fired up about [contraceptive access] because in … the Middle East for instance … they (physicians) really take strongly on the role of a parent and they worry so much about the future harm if women become sexually active and how they can help that child avoid it by giving very strict rules.”

Other examples centered around contraception and pregnancy, and women’s lack of autonomy in making decisions regarding pregnancy outcome.

#### Priority setting

Resource allocation was of interest to both students and faculty, specifically how to make decisions at both the patient and program level. Faculty identified this as a difficult subject to teach sensitively, as students’ backgrounds strongly affected their perception of priority issues:

“Like if you come from a particular country where you’re just barely getting basic needs met, for instance not even have sanitation and basic food and shelter then to hear people talking about, you know, putting money into really expensive end-of-life care to extend already privileged lives could just be like, ‘well gosh, what do you guys spend your time and money on over here?’ And vice-versa.”

#### Legal considerations

Several participants mentioned the conflict that can arise between legal and ethical precedents. They felt that legal norms trumped ethical considerations in order to avoid jeopardizing their medical careers or licenses:

“I think we do a good job of talking about your own feelings and views. I think what we’re missing is … what is expected of us and what are the legal consequences of that … cause otherwise people just kind of think that they’re allowed to do whatever they feel, versus knowing that sometimes your feelings are in conflict with the actual law.”

#### Cultural and religious bias

Culture and religion in decision-making were flagged as important, although the appropriateness of their influence on healthcare decision-making was questioned:

“We are very much imposed (to allowing) our cultural … views on the patient (to influence medical decision making) and this, in my view, shouldn’t be done at all.”

Despite concerns about the role of culture and religion, participants believed that impartiality was important in treating culturally diverse populations:

“[One] should look at [the patient] as a human being, it doesn’t matter what background, what culture, what colour, what language he speaks.”

## Discussion

Developing ethics curricula that cater to groups of individuals with diverse beliefs is a challenge. Working with medical students from different geopolitical and religious backgrounds, as well as with faculty, we explored the components needed in the development of such a program. IPEME constituted an ideal platform for examining this issue as the students hail from widely divergent geopolitical, cultural and religious backgrounds. Students overwhelmingly identified the need for, and utility of, a unique ethics curriculum, and mentioned truth-telling as a particularly relevant issue. Other topics included the role of gender, priority setting, and legal, cultural and religious considerations in clinical decision making. They clearly favored teaching methods that were interactive and minimized frontal, didactic instruction.

Many of the themes emerging from this study align with the International Commission on Education for the 21st Century (Delors Report) [13], and find common ground with other core ethics resources [14]. The Delors report highlights knowledge of self and others, appreciation of diversity and similarities among cultures, empathy and cooperative behavior, respect for other cultures and values, the ability to resolve conflict through dialogue, and working toward common objectives as key qualities. The United Nations Declaration of Human Rights stresses the importance of understanding, tolerance, and friendship [15]. In tandem with the stated health-through-peace goal of IPEME, students clearly demonstrated their interest in learning and understanding others’ cultural, religious and geopolitical presumptions and beliefs. The close interactions encouraged by IPEME’s unique make-up and the ready availability of qualified and sympathetic personnel may facilitate modelling in a very robust ‘hidden curriculum’ fashion [16, 17], the importance of which is only now being fully appreciated. Positive relationships and friendships have often sprung up spontaneously between participating students, leading to ongoing connections between alumni and cross-border visits.

### Limitations

This was a study based on a single cohort of medical students. Faculty views were incorporated into the study design to reflect lessons learned from previous groups of IPEME students. Interviewees' views may reflect what they thought researchers wanted to hear, although utilization of an interviewer unknown to students may have mitigated this effect, and parallel analysis of faculty provided some degree of verification of data. IPEME’s application process is designed to select students whose views accord with the health-for-peace philosophy, and this may skew the goals and beliefs reflected in their interviews. It is likely that lessons learned from our study will be helpful to others developing ethics curricula for diverse medical student groups.

Our findings enabled us to create a curriculum that was delivered to IPEME medical students the following year. Further study will be needed to assess the utility of implementing our suggestions, and this forms the basis of our ongoing research.

## Conclusions

Understanding the issues relevant, and common, to a diverse student body such as those exemplified by our students, enabled us to identify specific high impact topics to be used in bioethics instruction. The structure, content and delivery of an ethics curriculum within a brief medical elective necessitates the careful choice of both topics and modalities. Students’ clear choice of interactive instruction over frontal imparting of knowledge stresses the potential need to develop unique instructional modalities in order to reach some of the worthy goals aspired to by bioethicists.

To the best of our knowledge, this is the first study utilizing a needs analysis from medical students coming from different geopolitical and religious beliefs to explore the content and method of delivery of an ethics curriculum catering to the unique needs of students from different geopolitical regions. It provides suggestions and insights into the development of an ethics curriculum that could be used for a diverse student body.

## Endnotes

The University of Toronto Joint Centre for Bioethics is a partnership between the University of Toronto and affiliated healthcare organizations. It studies important ethical health-related topics through research and clinical activities. It consists of a network of over 180 multidisciplinary professionals seeking to improve health care standards at both national and international levels.

## Abbreviation

IPEME, The International Pediatric Emergency Medicine Elective

## Additional files

Additional file 1: Appendix I. Semi-structured interview guide for IPEME students.pdf. Outline of interview used with IPEME students. (DOC 25 kb) Additional file 2: Appendix II. Focus group guide for IPEME faculty.pdf. Outline of interview used with IPEME faculty. (DOC 24 kb) Additional file 3: Appendix III. Focus group guide for Joint Centre for Bioethics faculty.pdf. Outline of interview used for Joint Centre for Bioethics faculty. (DOC 30 kb)
