# Supplementary material for: Developing a bioethics curriculum for medical students from divergent geo-political regions
Source: BMC Med Educ. 2016 Jul 27;16:193. doi: 10.1186/s12909-016-0711-4 (PMC4962426; doi:10.1186/s12909-016-0711-4)
Supplement: Additional file 1: — Appendix I. Semi-structured interview guide for IPEME students.pdf. Outline of interview used with IPEME students. (DOC 25 kb) [file 12909_2016_711_MOESM1_ESM.doc]

**Semi-Structured Interview Guide for Students:**

1. From your perspective, what is ethics?
2. How is it relevant in health care?
   - 1. Is it being taught in your country’s health care context?
     2. Formally?
     3. Informally?
   1. How is it being taught?
      1. Could it be taught better?
3. What ethical issues have you encountered in your country’s health care context?
   1. Probes: (To bridge IPEME and general applicability)
      - 1. [see page 2 for possible probes]
4. Do you think discussions of ethics would be valuable in programs such as IPEME that cater to medical students from different religious, cultural, ethnic, and geographical areas?
   1. Why or why not?
   2. What would be some examples of clinical ethics issues that would be valuable to discuss during the IPEME program?
      1. What ethical issues relating to providing culturally sensitive care are relevant to the practice of medicine in your home country?
5. What ethical issues have arisen during the IPEME elective related to patient care?
   1. Please list the following ethical issues for the IPEME program in order from the most to least important:
      1. Truth-telling
      2. Confidentiality
      3. Resource allocation
      4. Diversity / Cultural Competence
      5. Priority setting
6. What would be the best teaching format(s) to deliver an IPEME ethics curriculum?
   1. Lectures, workshops, case studies, i-clicker, video, role play, etc?
      1. PROBES:
         1. Truth-telling
            1. Parent wishes that you do not to tell the patient about their illness
            2. Patient asks ‘what is wrong’ but parent(s) has requested patient not to know
         2. Confidentiality
            1. Reporting abuse or neglect
            2. Warning third-party persons of potential transmission of disease(s) i.e. HIV
            3. Psychiatric patient who you fear may harm himself or others
         3. Diversity
            1. Patient/ family requests for clinicians from the same part of the world, same skin colour, or requests that the clinician not be from the family’s community
            2. Language barriers between physician and patient:

Should you request a translator?

What should you do about a fear of miscommunication about illness, patient/family values, etc.

What if you realize that a translator is giving false or inaccurate translations to the patient / family regarding the patient’s illness or treatment options?

- - - - 1. Your/others moral/religious beliefs are at odds with hospital policy, or between physician views
        2. Respecting those that are different, or those with views or cultural beliefs that are different
      1. Assent /Role of Child as a Capable Decision Maker
         1. Conflict between family and patient regarding cessation of treatment
         2. One parent speaking for both regarding treatment decisions

What if there is a disagreement between the parents regarding treatment decisions?

- - - - 1. Treating a patient without consent

When parents cannot be reached to provide it

- - - 1. Resource Allocation

Should a child who needs a kidney transplant be sent out of the country if that country does not offer it?

How should these decisions be made?

- - - - 1. Should a child with resistance to a cheaper antibiotic get the more expensive one if that will affect the hospital’s global budget?
        2. Who should pay for health care?

Government

Private insurance policies

- - - 1. Sexuality and Sex issues
         1. You see a patient who you think is pregnant and she denies the possibility

To what point should you try to convince the patient of the possibility?

Should you perform a pregnancy test anyways?

- - - - 1. Patient requests an abortion

Should you tell her parents?

What if a patient does not want abortion, but parents insist she has one?

- - - - 1. If a Patient reveals their sexual orientation as LGBT, can you as a physician refuse to treat him or her?
      1. Organ transplants / donation
         1. Family refuses critical organ transplant on religious/cultural grounds
         2. Family requests to know about the organ donor to go forward with the transplant
