# Supplementary material for: Developing a bioethics curriculum for medical students from divergent geo-political regions
Source: BMC Med Educ. 2016 Jul 27;16:193. doi: 10.1186/s12909-016-0711-4 (PMC4962426; doi:10.1186/s12909-016-0711-4)
Supplement: Additional file 3: — Appendix III. Focus group guide for Joint Centre for Bioethics faculty.pdf. Outline of interview used for Joint Centre for Bioethics faculty. (DOC 30 kb) [file 12909_2016_711_MOESM3_ESM.doc]

**Focus Group Guide for Joint Centre for Bioethics Faculty**

1. Do you think discussions of ethics would be valuable in a program such as IPEME? Why or why not?
   1. What ethical issues do you think would be valuable to discuss during the IPEME program?
2. What ethical issues have arisen in providing education to cross border and culturally diverse groups in healthcare?
   1. Please list the following ethical issues for the IPEME program in order from the most to least important:
      1. Truth-telling
      2. Confidentiality
      3. Resource allocation
      4. Diversity / Cultural Competence
      5. Priority setting
3. What would be the best teaching formats to deliver an IPEME ethics curriculum? Lectures, workshops, case studies, i-clicker, video, role play etc?
